# The Impact of Hospital Volume on Postoperative Outcomes for Esophagectomy and Gastrectomy: A Systematic Review and Meta-analysis

**DOI:** 10.1245/s10434-026-19558-5

**Published:** 2026-04-01

**Authors:** Cezanne D. Kooij, Irene S. Zuin, Alexandre Challine, Jessie A. Elliott, Jelle P. Ruurda, Richard van Hillegersberg, Lucas Goense

**Affiliations:** 1 University Medical Center Utrecht, Utrecht, The Netherlands; 2 Department of Digestive Surgery, Sorbonne Université, AP-HP, Hôpital Saint Antoine, Paris, France; 3 Department of Surgery, Trinity St. James’s Cancer Institute, Dublin, Ireland

**Keywords:** Esophagectomy, Gastrectomy, Esophagogastric surgery, Hospital volume, Postoperative outcomes, Centralization

## Abstract

**Background:**

High-volume hospitals tend to have better outcomes in complex surgeries, but outcome variations and volume thresholds complicate conclusions. This systematic review/meta-analysis assessed the impact of hospital volume on postoperative outcomes after esophagogastric surgery and identified volume thresholds beyond which benefits plateau.

**Methods:**

PubMed/MEDLINE was searched for cohort studies (2013–2023) on volume and outcomes after esophagectomy/gastrectomy for cancer. Primary outcome was 30-day mortality. Secondary outcomes included 90-day mortality, complications, length of stay (LOS), and long-term survival. The meta-analysis compared hospital volumes using odds ratios (ORs) for binary outcomes, hazard ratios (HRs) for survival, and mean differences (MDs) for LOS. A generalized estimating equation model assessed the continuous association between volume and 30-day mortality. Segmented regression identified volume thresholds where outcomes plateaued.

**Results:**

Of 2679 articles, 56 studies on hospital volume and outcomes after esophagectomy and gastrectomy for cancer were included. High-volume hospitals (4–239 resections/year) showed lower 30-day mortality (OR 0.51; 95% confidence interval [CI] 0.43–0.59), 90-day mortality (OR 0.65; 95% CI 0.56–0.74), fewer complications (OR 0.83; 95% CI 0.74–0.94), shorter LOS (MD -1.50 days; 95% CI 0.97–2.03), and better survival (HR 0.83; 95% CI 0.78–0.87). Each doubling of volume demonstrated a significant reduction in 30-day mortality for esophagectomy (OR/volume-doubling 0.74; 95% CI 0.68–0.81) and gastrectomy (OR/volume-doubling 0.70; 95% CI 0.61–0.82). Breakpoints were identified at 43 cases for esophagectomy and 15 cases for gastrectomy per year, beyond which the association plateaued.

**Conclusions:**

Higher hospital volume is associated with lower mortality, reduced complications, shorter LOS, and improved survival. Identified thresholds exceed existing policy benchmarks, supporting further centralization of esophagogastric cancer surgery.

**Supplementary Information:**

The online version contains supplementary material available at 10.1245/s10434-026-19558-5.

Esophagogastric cancer continues to pose a significant global health burden in terms of cancer-related mortality.^1^ Despite advancements in the treatment of esophageal and gastric cancers in recent decades, surgery remains the cornerstone of managing these malignancies.^2^ However, surgical morbidity and mortality with these complex surgical procedures remains relatively high, with considerable differences between healthcare institutions.

The landmark paper by Luft et al.^3^ first suggested a potential association between hospital volume and improved mortality rates for complex surgical procedures. Since then, numerous studies have evaluated this concept in esophagectomy and gastrectomy. Many have shown that multidisciplinary care in high-volume hospitals improves postoperative mortality rates after esophagectomy and gastrectomy.^4–6^ However, the varied thresholds for defining high-volume hospitals, and differences in the study outcomes assessed has created a fragmented body of evidence. Although early influential studies based on national datasets introduced volume categories, these were generally derived by broadly grouping hospitals into volume strata.^7^ Consequently, no universally accepted threshold for defining high-volume centers exists. Reported thresholds vary widely across studies, complicating efforts to establish evidence-based standards for centralizing care.

Given these gaps, a comprehensive analysis of recently published literature will provide valuable insights into the effect of hospital volume on patient outcomes, beyond mortality alone. This study aimed to meta-analyze the volume–outcome relationship for esophagectomy and gastrectomy, examining a range of postoperative outcomes, including mortality, complications, and long-term survival. In addition, stratified data from the literature were evaluated to determine an optimal volume threshold.

## Materials and Methods

This systematic review was conducted in accordance with the Preferred Reporting Items for Systematic Reviews and Meta-Analyses (PRISMA) guidelines.^8^ The study is registered to PROSPERO with the ID CRD42024570165.

### Search Strategy

A systematic PubMed/MEDLINE search was conducted on May 10, 2024, using medical subject heading (MeSH) terms and Boolean operators: “((esophagectomy) OR (gastrectomy) OR (oesophagectomy)) AND ((volume) OR (caseload))”. To mitigate outcome variability due to advancements in surgical techniques and technologies, a retrospective analysis covering a 10-year period was employed (2013–2023).

### Study Selection and Inclusion Criteria

The PICO (patients/population, intervention, comparison, outcome; Table S1) framework guided the selection criteria. After removing duplicates, one author (A.C.) screened titles and abstracts, then two authors (I.S.Z. and C.D.K.) assessed the full text. Included were full-text prospective/retrospective (human) cohort studies on hospital volume and outcomes after esophageal or gastric surgery for cancer, published in English. Excluded were systematic reviews, case reports/series, pediatric studies, and conference abstracts.

### Data Extraction

Data collection was conducted using a standardized data extraction form, with one author (I.S.Z.) performing the extraction and another (C.D.K.) verifying the extracted information. Extracted variables included the first author, year of publication, study design, hospital characteristics, number of patients and centers, volume thresholds, and outcome measures. In cases of overlapping patient populations from the same authors or research groups, studies with the largest patient cohorts were prioritized for inclusion in the meta-analysis, and studies with partially overlapping populations from different groups were included if their populations differed in period or inclusion criteria.

### Outcomes

The primary outcome was 30-day mortality. Secondary outcomes included 90-day mortality, overall postoperative complication rate, anastomotic leakage, pulmonary complications, length of stay (LOS), readmission rate, and long-term survival.

### Risk of Bias and Quality Assessment

The quality of the observational studies was assessed independently by two reviewers (I.S.Z and C.D.K.), using the New Ottawa Scale, a validated tool for assessing risk of bias in non-randomized studies.^9^

### Data Synthesis and Meta-Analysis

For meta-analysis, outcomes were compared between high-volume and low-volume hospitals, with volume categories defined according to each included study. If multiple volume thresholds were used within a study, the comparison between the lowest and highest threshold was included. Multiple meta-analyses were conducted based on studies' reported crude estimates, grouping the studies according to the postoperative outcomes tested. Binary outcomes were analyzed usings odds ratios (ORs), studies reporting risk ratios were recomputed into ORs using raw data. Mean differences in LOS were calculated, with standard errors estimated from reported means and standard deviations. For survival outcomes, hazard ratios (HRs) with 95% confidence intervals (CIs) were extracted. If only Kaplan–Meier curves were available, HRs and 95% CIs were estimated based on the data extracted with WebPlotDigitizer 4.6, following the method proposed by Tierney et al.^10^

For all outcomes, pooled estimates were computed using a random-effects model, assuming clinical heterogeneity among the included studies. Results from esophagectomy and gastrectomy studies were combined for an overall estimate. Findings were reported independently only if subgroup analysis according to surgical procedure (esophagectomy and gastrectomy) showed a significant interaction with outcomes. The presence of statistical heterogeneity across studies was assessed using the I^2^ statistic. Potential sources of heterogeneity were assessed by adding the following study characteristics as covariates to the model: geographic region (East vs West), quality of study (moderate vs high quality), and multivariable correction of primary outcome.

For the primary analysis, ORs derived from dichotomized data were used to assess the impact of low- versus high-volume hospitals on postoperative outcomes. However, dichotomizing a continuous variable such as hospital volume results in significant data loss, limiting accurate identification of optimal volume thresholds. To overcome this limitation, a secondary analysis was performed to investigate the continuous association between hospital volume and 30-day mortality. No other outcomes were assessed because the number of available studies was too limited for meaningful analysis. Raw data were extracted from included studies to estimate the risk of mortality across different volume strata. Only studies providing sufficient data on volume cut-offs, mortality rates within each category, and the number of patients per category were included in this analysis. The mean volume for each stratum was estimated using the midpoint between the upper and lower limits. If the upper limit was not reported, it was approximated as twice the lower limit, based on observed data patterns. Hospital volume was transformed using a log base 2 scale to address skewness from a high number of low-volume categories and to reflect a multiplicative relationship in volume effects.

A generalized estimating equations (GEE) model was used to plot mortality rates in each stratum against hospital volume, accounting for within-study clustering to address the non-independence of volume strata within the same study. This analysis yielded an OR that estimated the change in mortality for each doubling of volume. A segmented regression model was used to assess whether there was a statistically significant breakpoint in the association between hospital volume and mortality. This method identifies whether there is a threshold beyond which the relationship between volume and mortality plateaus or changes. Once a breakpoint was identified, it was incorporated into the GEE model to evaluate the population-averaged effects. A significant difference in slope before and after the breakpoint in the GEE model confirms a threshold effect, indicating a change in the volume–mortality association from this point.

Publication bias was evaluated visually for the primary outcome (30-day mortality) by inspecting funnel plots and statistically through Egger’s regression test.^11^ All P-values were considered significant if P<0.05. All statistical analysis were performed using R (version 4.0.0 R Project for Statistical Computing).

## Results

**Fig. 1:**
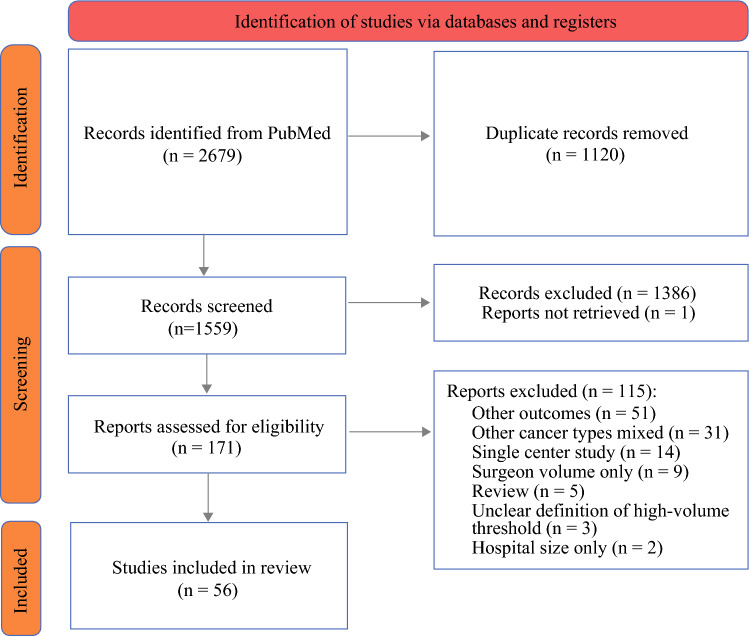
Preferred Reporting Items for Systematic Reviews and Meta-Analyses (PRISMA) flowchart of study selection

The initial search identified 2679 studies. After the removal of duplicates, a total of 1559 articles were screened based on title and abstract for eligibility. Of these, the full-text of 171 relevant articles were assessed, of which 115 articles were excluded. Figure 1 displays the PRISMA flowchart and provides further details on the exclusion/inclusion process. Eventually, 56 studies were included in this systematic review and meta-analysis.^4–6,12–64^

### Characteristics of Included Studies

The characteristics of the 56 included studies are summarized in Tables 1 and 2. According to the risk-of-bias assessment (Table S2), eight articles were classified as moderate quality (4–6 points), and the other 48 articles were classified as high quality (7–9 points). The number of patients in the included studies ranged from 908^57^ to 158,618^4^ for esophagectomy and from 88^33^ to 145,523^36^ for gastrectomy. Two studies were prospective,^20,23^ and the remaining studies were retrospective cohort studies. In total, 41 studies were conducted in Western countries, 16 studies in Eastern countries, and two had a global scope. The most frequently used high-volume hospital cut-off was 20 resections per year (*n* = 7 [12.5%]), followed by 40 resections per year (*n* = 6 [10.7%]). The lowest threshold for high volume was defined as four and five resections per year for esophagectomy and gastrectomy, respectively, and the highest thresholds were 87 and 239. The most commonly used primary outcome was 30-day mortality (*n* = 18 [32.1%]).

**Table 1:** Characteristics of studies that analyzed the relationship between hospital volume and outcomes following esophagectomy

Study	Country	Study design	Period	Patients (*n*)	Hospitals (*n*)	Volume cut-off (cases/year)	Primary outcome
Adu-Gyamfi et al.^53^	USA	Retrospective	2013	2017	NS	10	30-day readmission rate
Arnold et al.^13^	USA	Retrospective	2004–2012	5580	663	13	90-day mortality
Clark et al.^21^	USA	Retrospective	2007–2013	4330	156	20	In hospital mortality
Coupland et al.^22^	England	Retrospective	2004–2008	13,189	144	20^a^	Resection rate and survival
D'Journo et al.^23^	Australia, Belgium, Brazil, Canada, China, Denmark, France, Germany, India, Ireland, Italy, Japan, Netherlands, Singapore, Spain, Sweden, Switzerland, UK, USA	Prospective	2015–2019	8640	39	71.7^a^	90-day mortality
Fumagalli et al.^25^	Italy	Retrospective	2005–2011	2801	111	21^a^	30-day mortality
Gabriel et al.^26^	USA	Retrospective	2004–2013	17,547	NS	20^a^	Hospital LOS, readmission rate, 30- and 90-day mortality
Gandjian et al.^27^	USA	Retrospective	2010–2018	23,176	Changes in time	20	Mortality
Habbous et al.^28^	Canada	Retrospective	2010–2018	10,364	NS	20^a^	Overall survival
Henneman et al.^29^	The Netherlands	Retrospective	1989–2009	10,025	44	20	6-months and 2-years mortality
Holleran et al.^30^	USA	Retrospective	2008–2019	2212	NS	4	30-day mortality
Hsu et al.^31^	Taiwan	Retrospective	2008–2011	2152	58	22	30-day mortality
Hue et al.^32^	USA	Retrospective	2010–2016	1565	212	9	90-day mortality
Jafari et al.^38^	USA	Retrospective	2001–2010	15,290	NS	10	Morbidity rate and in hospital mortality
Kennedy et al.^41^	USA	Retrospective	2004–2013	9270	NS	87^a^	In hospital mortality
Kim et al.^5^	Korea	Retrospective	2004–2017	11,346	122	48^a^	In hospital mortality
Lei et al.^4^	China	Retrospective	1973–2020	158,618	139	41^a^	In hospital mortality
Markar et al.^45^	French speaking European centers	Retrospective	2000–2010	2944	30	8	In hospital and 30-day mortality
Meng et al.^46^	New Zealand	Retrospective	2008–2015	2252	65	11^a^	In hospital and 30-day mortality
Munasinghe et al.^47^	UK and USA	Retrospective	2005–2010	7433 UK; 5858 USA	66 UK; 775 USA	26.4	In hospital mortality
Narendra et al.^49^	Australia	Retrospective	2001–2015	1167	24	6	Textbook outcome
Nishigori et al.^51^	Japan	Retrospective	2011–2013	16,556	988	30^a^	30-day mortality
Nuytens et al.^52^	France	Retrospective	2017–2019	2675	18	5	90-day mortality
Patel et al.^54^	USA	Retrospective	2006–2013	11,739	1018	20	Overall survival
Salfity et al.^55^	USA	Retrospective	2010–2013	8656	NS	42.5	30- and 90-day mortality
Smith et al.^57^	Australia	Retrospective	200–2009	908	42	6	LOS
Ubels et al.^58^	Australia, Belgium, Brazil, Colombia, Finland, France, Germany, Greece, Italy, Ireland, Libya, Netherlands, Pakistan, Portugal, Romania, Spain, Sweden, Switzerland, Turkey, UK	Retrospective	2011–2019	1509	71	60^a^	Failure to rescue
Voeten et al.^62^	The Netherlands	Retrospective	2016–2019	3135	16	40	Morbidity rate
Voeten et al.^61^	The Netherlands	Retrospective	2011–2018	6172	25–19	40	LOS
Voeten et al.^60^	The Netherlands	Retrospective	2015–2018	1007	17	40	Textbook outcome
Voeten et al.^59^	The Netherlands	Retrospective	2011–2018	5894	22	40^a^	Failure to rescue
Yoshida et al.^63^	Japan	Retrospective	2012–2016	24,223	1002	43.2^a^	30-day mortality

LOS, length of stay; NS, not specified

^a^Studies with multiple volume cut-offs

**Table 2:** Characteristics of studies that analyzed the relationship between hospital volume and outcomes following gastrectomy

Study	Country	Study design	Period	Patients (*n*)	Hospitals (*n*)	Volume cut-off (cases/year)	Primary outcome
Altini et al.^12^	Italy	Retrospective	2004–2008	1314	16	21^a^	30-day mortality
Asplund et al.^22^	Sweden	Retrospective	2006–2020	1774	60	15.7^a^	5-year mortality
Busweiler et al.^15^	The Netherlands	Retrospective	2005–2014	4837	NS	40^a^	Lymph node yield and 30-day mortality
Challine et al.^16^	France	Retrospective	2013–2018	10,343	661	24^a^	In-hospital mortality
Choi et al.^17^	Korea	Retrospective	2004–2013	2550	704	30^a^	Cancer-specific mortality
Cibulas et al.^18^	USA	Retrospective	2004–2016	34,688	NS	38^a^	Textbook outcome
Claassen et al.^20^	The Netherlands, Sweden, Denmark	Prospective	2007–2015	788	56	21	Overall survival
Claassen et al.^20^	The Netherlands	Retrospective	2007–2015	494	NS	30^a^	Lymph node yield and resection rate
Coupland et al.^22^	England	Retrospective	2004–2008	13,189	144	20^a^	Resection rate and survival
Diers et al.^24^	Germany	Retrospective	2009–2017	46,187	1.084	30^a^	In-hospital mortality
Gabriel et al.^26^	USA	Retrospective	2004–2013	20,059	NS	20^a^	Hospital LOS, readmission rate, 30- and 90-day mortality
Ichikawa et al.^33^	Japan	Retrospective	2007–2013	88	11	100	Morbidity rate
Ichikawa et al.^34^	Japan	Retrospective	2002–2012	420	17	100	Overall survival
Ikoma et al.^35^	USA	Retrospective	2010–2015	2733	NS	15^a^	In-hospital mortality
Iwatsuki et al.^36^	Japan	Retrospective	2011–2015	145,523	2182	52^a^	30-day mortality
Iwatsuki et al.^37^	Japan	Retrospective	2011–2015	71,307	2,051	27^a^	30-day mortality
Ji et al.^39^	China	Retrospective	2013–2018	125,683	515	239^a^	In-hospital mortality
Ju et al.^40^	USA	Retrospective	2004–2015	29,559	1144	17	Overall survival
Lacueva et al.^42^	Spain	Retrospective	2013–2016	591	17	10	Morbidity rate
Lee et al.^43^	Korea	Retrospective	NS	284	3	100	Perioperative outcomes
Levy et al.^44^	Canada	Retrospective	2004–2015	1660	69	12	Textbook outcome
Murata et al.^6^	Japan	Retrospective	2009–2011	12,522	741	40	In-hospital mortality
Narendra et al.^48^	Australia	Retrospective	2001–2015	1080	49	5	Textbook outcome
Narendra et al.^49^	Australia	Retrospective	2001–2015	1266	48	5	30- and 90-day mortality
Nimptsch et al.^50^	Germany	Retrospective	2010–2015	72,528	NS	28^a^ (50)	In-hospital mortality
Salfity et al.^55^	USA	Retrospective	2010–2013	8656	NS	42.5	30- and 90-day mortality
Shibao et al.^56^	Japan	Retrospective	2012–2013	37,752	1,074	62^a^ (98)	30- and 90-day mortality
Smith et al.^57^	Australia	Retrospective	200–2009	1621	84	6	LOS
Voeten et al.^61^	The Netherlands	Retrospective	2011–2018	3690	42–20	40	Textbook outcome
Wu et al.^64^	Taiwan	Retrospective	2000-2010	7905	NS	10	30-day mortality

LOS, length of stay; NS, not specified

^a^Studies with multiple volume cut-offs

### 30-Day Mortality and Threshold Analysis

**Fig. 2:**
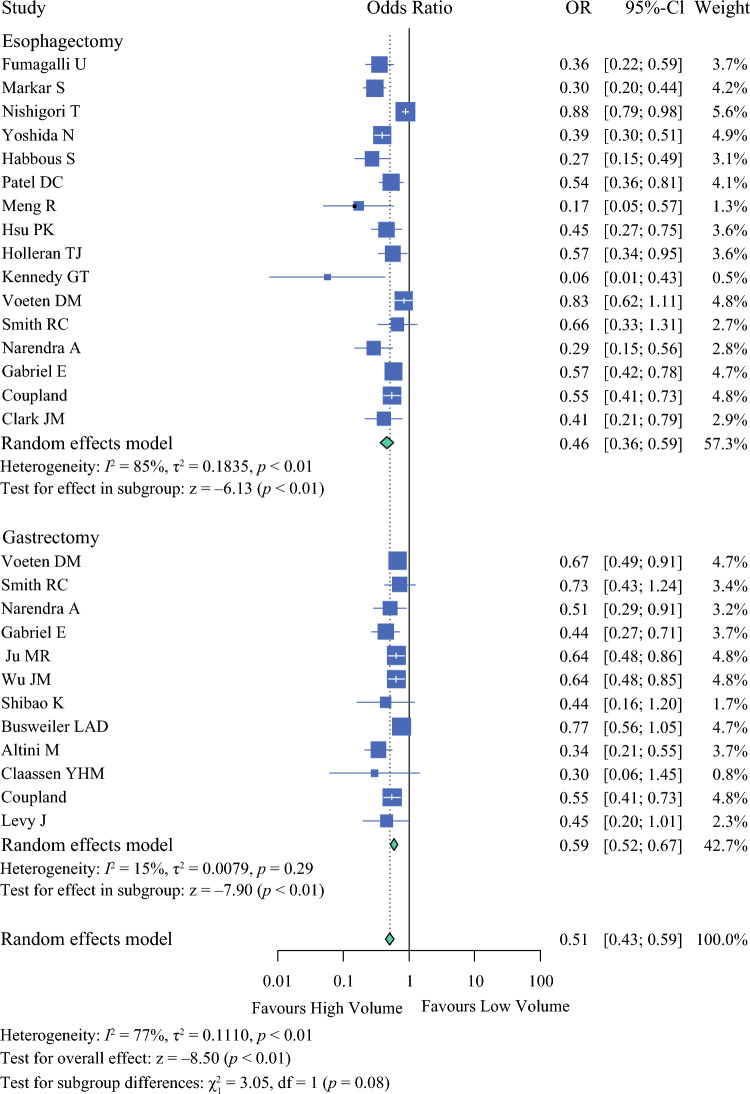
Meta-analysis of the relationship between 30-day mortality and hospital volume for esophagectomy and gastrectomy. CI, confidence interval; OR, odds ratio

Of the 54 included studies, 28 reported 30-day postoperative mortality, with 16 focusing on esophagectomy^21,22,25,26,28,30,31,41,45,46,49,51,54,57,61,63^ and 12 on gastrectomy.^12,15,19,22,26,40,44,48,56,57,61,64^ The pooled OR was 0.51 (95% CI 0.43–0.59; *I*^2^ = 77%) favoring high-volume hospitals. Subgroup analysis revealed no significant difference between esophagectomy and gastrectomy (Fig. 2). Egger's regression test (*P* < 0.001) and the funnel plot (Fig. S1) indicated potential publication bias, with a clustering of studies reporting significant ORs on the left side of the plot, suggesting a preference for publishing positive findings. There were no significant differences in the heterogeneity of 30-day mortality based on geographic region (*P* = 0.65), quality of study (*P* = 0.43), or multivariable correction (*P* = 0.65).

**Fig. 3:**
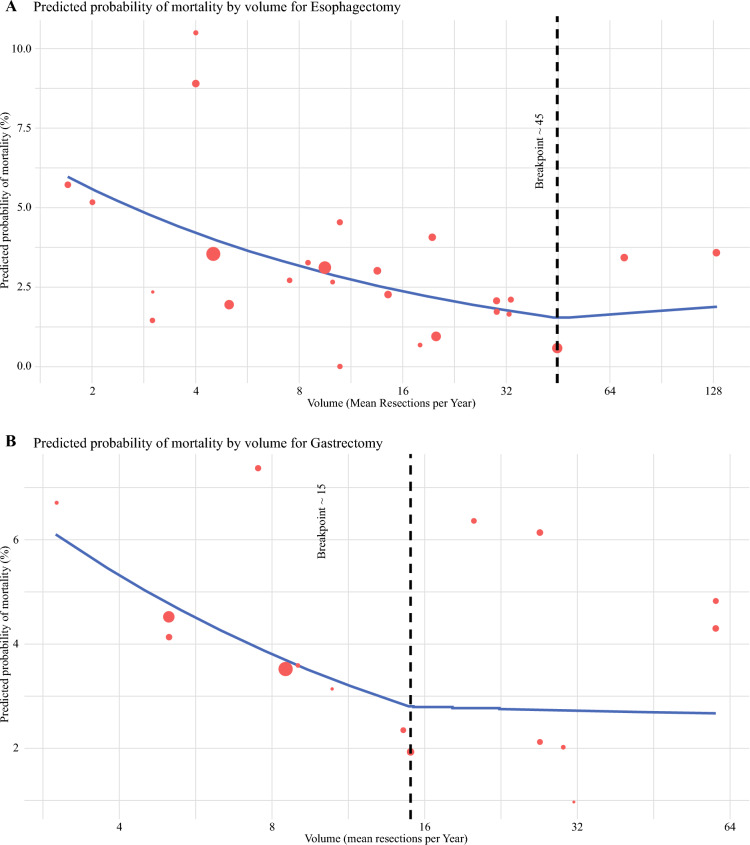
The generalized estimating equations model of the predicted mortality probability against hospital volume, with a segmented regression identifying the breakpoints in the volume–mortality relationship for esophagectomy (**A**) and gastrectomy (**B**)

The threshold analysis, shown in Fig. 3, revealed that each doubling of hospital volume was associated with a significant reduction in 30-day mortality rate for both esophagectomy (OR per volume doubling: 0.74; 95% CI 0.68–0.81) and gastrectomy (OR per volume doubling: 0.70; 95% CI 0.61–0.82).

Segmented model analysis identified a breakpoint at 43 resections per year for esophagectomy and 15 per year for gastrectomy, beyond which the volume–mortality association reached a plateau.

### 90-Day Mortality

In total, 14 of 54 studies reported 90-day mortality, eight for esophagectomy^13,23,26–28,52,54,57^ and six for gastrectomy.^14,26,40,48,56,57^ The pooled OR was 0.65 (95% CI 0.56–0.74; *I*^2^ = 0.66) favoring high-volume hospitals, for both esophagectomy and gastrectomy (*P*-value subgroup difference = 0.70). Results are shown in Fig. S2.

### Postoperative Complications

Among the 54 studies, 13 reported overall complication rates, four for esophagectomy^30,38,53,61^ and nine for gastrectomy.^6,20,24,34,35,43,44,50,61^ The pooled OR for the association between hospital volume and overall complication rates was 0.83 (95% CI 0.74–0.94; *I*^2^ = 0.91) favoring high-volume hospitals for both esophagectomy and gastrectomy (*P*-value subgroup difference = 0.42). Results are shown in Fig. S3.

### Pulmonary Complications

Of the eight studies reporting on pulmonary complications, six focused on esophagectomy^27,30,38,45,58,61^ and two on gastrectomy.^20,61^ The pooled OR was 0.79 (95% CI 0.62–0.1.00; *I*^2^ = 93%), for both esophagectomy and gastrectomy favoring high-volume hospitals (*P*-value subgroup difference = 0.52). Results are shown in Fig. S4.

### Anastomotic Leakage

Of the six studies reporting on anastomotic leakage, three focused on esophagectomy^38,45,61^ and three on gastrectomy.^20,24,61^ There was no sign of a significant association between anastomotic leakage and hospital volume, with a pooled OR of 1.00 (95% CI 0.58–1.70; *I*^2^ = 97%) for both esophagectomy and gastrectomy (*P*-value subgroup difference = 0.22). Results are shown in Fig. S5.

### LOS

Of the 17 studies reporting on the association between hospital volume and LOS, 10 focused on esophagectomy^26,27,30,32,38,46,47,53,54,58^ and seven on gastrectomy.^24,26,35,43,44,50,64^ The pooled LOS was 1.5 days shorter (95% CI 0.97–2.03; *I*^2^ = 97%) in high-volume hospitals than in low-volume hospitals, for both esophagectomy and gastrectomy (*P*-value subgroup difference = 0.75). Results are shown in Fig. S6.

### Readmissions

Of the 16 studies reporting on readmission, 10 focused on esophagectomy^13,26,27,32,46,49,53,54,57,61^ and six on gastrectomy.^26,40,44,49,57,61^ The readmission rate showed no significant difference between high- and low-volume hospitals, with an OR of 0.92 (95% CI 0.79–1.06) for both esophagectomy and gastrectomy (*P*-value subgroup difference = 0.93). Results are shown in Fig. S7.

### Long-Term Survival

An analysis of overall survival in relation to hospital volume was performed using HRs from eight studies on esophagectomy^4,5,22,26,31,49,54,57^ and six studies on gastrectomy^12,19,22,26,49,57^ (Fig. S8). The results indicate that patients who underwent surgery in high-volume hospitals had significantly better overall survival than those treated in low-volume centers (OR 0.83; 95% CI 0.78–0.87), for both esophagectomy and gastrectomy (*P*-value subgroup difference = 0.35).

## Discussion

This systematic review and meta-analysis offers a comprehensive overview of the association between hospital volume and short- and long-term postoperative outcomes after esophagectomy and gastrectomy for cancer. Although previous reviews have explored the volume–outcome association for specific postoperative outcomes, this study distinguishes itself by integrating multiple postoperative outcomes, including 30- and 90-day mortality, postoperative complication rates, LOS, readmission rate, and long-term overall survival. This approach offers a more holistic view of the impact of hospital volume on patient care, from the immediate postoperative period to long-term survival. By including volume–outcome data from global healthcare systems, the present study provides insights into the impact of hospital volume on postoperative outcomes that have a high degree of external validity, which may inform the design of healthcare networks and specialist services internationally. Importantly, the current study identified procedural volume thresholds (43 cases for esophagectomy and 15 cases for gastrectomy per year), beyond which the association between procedural volume and 30-day mortality plateaus.

Previous studies and meta-analyses have reported a significant association between hospital volume and postoperative mortality, especially for complex surgical procedures.^3,7^ Consequently, efforts have been made at a national level to centralize these high-risk procedures, with varying degrees of success in countries such as the United Kingdom, Ireland, Canada, and the Netherlands.^65^ However, global guidelines on centralization remain inconsistent. A recent questionnaire in Western Europe revealed considerable variation in centralization practices for esophagectomy. In some countries (31%), specific volume thresholds were in place, ranging from 10 to 26 procedures per year. Others (44%) had centralized care through designated centers, and the remaining countries (25%) had no centralization policies in place.^66^ Earlier reviews attempting to define optimal thresholds were limited by methodological constraints, including reliance on arbitrary dichotomization of volume data or linear trend analyses incorporating older studies.^67–69^ For instance, these older studies included centers performing only one to two esophagectomies annually, a practice increasingly rare in current healthcare practice. The current study mitigates the influence of historic low-volume practices by focusing on more recent literature, better reflecting current surgical volumes.

Furthermore, the segmented regression analysis represents a methodological advancement over prior approaches, as it avoids the loss of information inherent in dichotomizing continuous volume data and accounts for within-study clustering. This approach identified plateaus in the volume–outcome relationship at 43 cases for esophagectomy and 15 cases for gastrectomy per year, potentially informing real-world centralization benchmarks. These values should not be interpreted as strict or binary thresholds, nor as implying that outcomes below these levels are poor or that outcomes above them are categorically superior. Rather, they represent evidence-based indicators of diminishing benefit within a continuous volume–outcome relationship. From a health system perspective, these values may be interpreted as benchmarks indicating the range up to which further centralization is most likely to yield meaningful improvements in postoperative outcomes, particularly in regions where procedures are still distributed across low-volume centers. As such, these values are intended to inform policy decisions on regionalization and referral structures rather than define rigid institutional performance standards. Nevertheless, these thresholds need to be contextualized within international healthcare systems and considered within the context of the study’s limitations. For example, not all studies provided sufficient data for the segmented regression, and case-mix was not accounted for in the analysis. Very high-volume centers may increasingly manage more complex cases referred from other hospitals, which could affect their postoperative outcomes, potentially underestimating the benefits of high-volume settings. Despite these limitations, the established thresholds provide valuable insights to guide the centralization of esophagogastric cancer care.

This meta-analysis highlights the significant benefits of performing esophagectomy and gastrectomy for cancer in high-volume centers, including reduced morbidity and improved short- and long-term survival. These advantages likely stem from systemic factors that extend beyond procedural volume alone, such as surgeon and multidisciplinary team experience, multidisciplinary support structures, specialized nursing, and hospital infrastructure, including dedicated intensive care units and high nurse-to-patient ratios.^50,70–72^ However, these elements are often underreported or inadequately accounted for in volume–outcome studies, making their precise contribution to patient outcomes difficult to quantify. High-volume centers typically provide these resources, which likely contribute to improved survival rates, possibly because complications less often result in death, a concept known as failure to rescue, as well as shorter LOS. This further supports the case for cost-effective centralization. Future research should focus on quantifying institutional factors, including failure to rescue, to refine volume thresholds and ensure that centralization strategies optimize not only caseloads but also the expertise and support systems integral to high-quality surgical care.^49,69,72^

Although centralization of esophagectomy and gastrectomy has demonstrated clear benefits, it is important to consider the possibility of diminishing returns at very high procedural volumes. Although some studies report continued improvement in outcomes with increasing hospital volume,^62^ Vonlanthen et al.^73^ noted that the volume–outcome relationship may eventually plateau – or even decline – once a certain threshold is exceeded, potentially due to overstretched hospital infrastructure. Excessive caseloads may strain available resources, including operating room capacity, intensive care units, and specialized personnel, potentially leading to longer wait times, reduced individualized care, and an increased risk of complications. Therefore, policies should ensure that increasing surgical volume is accompanied by proportional investments in staffing, infrastructure, and multidisciplinary expertise to maintain high standards of care. Additionally, selective referral of more complex cases to high-volume centers may play a role in this dynamic, which could affect overall outcomes. However, the limited number of studies including centers performing over 100 procedures annually restricts the ability to fully evaluate the true effect of centralization. Many studies define "high-volume" as 20–40 cases per year, which, while beneficial compared with lower volumes, may not capture the potential advantages of further centralization. Future research should explore these upper limits to determine whether certain thresholds represent optimal, rather than maximal caseloads for achieving optimal outcomes.

This study has several limitations that should be acknowledged. First, the lack of standardized volume cut-offs across studies complicates the establishment of evidence-based thresholds. Although the segmented regression model helps mitigate this issue, substantial heterogeneity remains. The differences in geographic regions, surgical techniques, and treatment approaches may affect the external validity of the identified volume thresholds within individual healthcare systems. Also, the inconsistent reporting of surgical approaches (open vs minimally invasive surgery) in the included studies prevented assessment of their potential influence on outcomes.

Second, these thresholds do not account for potential differences in case complexity between high- and low-volume hospitals. High-volume centers often manage more complex or high-risk cases, which could diminish the observed volume–outcome association and influence the plateau effect. Many studies included in this meta-analysis did not adjust for baseline patient characteristics or case complexity, potentially underestimating the true benefits of high-volume centers.

Third, some studies used nationwide databases with overlapping periods. To minimize duplication, the largest cohorts from the same authors were prioritized for inclusion in the meta-analysis. Studies from different groups were retained when populations differed in period or inclusion criteria. This approach balanced potential overlap with the inclusion of relevant data. Furthermore, key variables, such as surgeon volume and the availability of dedicated nursing staff, were inconsistently reported, limiting the ability to assess their impact on outcomes. However, existing evidence suggests that hospital volume is more strongly associated with outcomes than surgeon volume, which may partially offset this limitation.^7,67^

In conclusion, this systematic review and meta-analysis demonstrates that higher hospital volume is associated with significantly lower postoperative mortality, fewer complications, shorter hospital stay and improved long-term survival for patients undergoing esophagectomy and gastrectomy for cancer. The identified volume thresholds exceed many existing policy benchmarks and support further centralization of esophagogastric cancer care and may inform updated volume standards.

## Supplementary Information

Below is the link to the electronic supplementary material.

Supplementary file1 (DOCX 1125 KB)

## Data Availability

Data used for analysis are available on request by emailing the corresponding author at l.goense@umcutrecht.nl.
