# Supplementary material for: The Impact of Hospital Volume on Postoperative Outcomes for Esophagectomy and Gastrectomy: A Systematic Review and Meta-analysis
Source: Ann Surg Oncol. 2026 Apr 1;33(8):7088–100. doi: 10.1245/s10434-026-19558-5 (PMC13337930; doi:10.1245/s10434-026-19558-5)
Supplement: Supplementary file 1 — Supplementary file1 (DOCX 1125 KB) [file 10434_2026_19558_MOESM1_ESM.docx]

**The Impact of Hospital Volume on Postoperative Outcomes for Esophagectomy and Gastrectomy: A Systematic Review and Meta-Analysis**

Cezanne D. Kooij MD^1^, Irene S. Zuin^1^, Alexandre Challine^2^, Jessie A. Elliott^3^, Jelle P. Ruurda^1^, Richard van Hillegersberg^1^, Lucas Goense^1^

^1^University Medical Center Utrecht, Utrecht, The Netherlands

^2^Department of Digestive Surgery, AP-HP, Hôpital Saint Antoine, F-75012, Paris, France; Sorbonne Université, France

^3^Department of Surgery, Trinity St. James’s Cancer Institute, Dublin, Ireland

**Corresponding author.** Lucas Goense, University Medical Center Utrecht, Heidelberglaan 100, 3584 CX Utrecht, The Netherlands, [L.Goense@umcutrecht.nl](mailto:L.Goense@umcutrecht.nl)

**Supplementary Materials - Index**

| **Supplementary Figures and Tables** |  |
| --- | --- |
| Table S1 | *pag. 2* |
| Table S2 | *pag. 3-5* |
| Figure S1 | *pag. 6* |
| Figure S2 | *pag. 7* |
| Figure S3 | *pag. 8* |
| Figure S4 | *pag. 9* |
| Figure S5 | *pag. 10* |
| Figure S6 | *pag. 11* |
| Figure S7 | *pag. 12* |
| Figure S8 | *pag. 13* |

**Supplementary Figures and Tables**

**Table S1.** The PICO (Population, Intervention, Comparison, Outcome) framework outlined for the research question regarding the impact of hospital volume on patient outcomes for esophagectomy and gastrectomy in the context of esophageal cancer.

| **P** | Population | What is the condition or disease of interest? | Patients with esophagogastric cancer |
| --- | --- | --- | --- |
| **I** | Intervention | What are the actions taken to intervene? | Esophagogastric surgery |
| **C** | Comparison | What is/are the comparison or alternative treatment(s)? | High-volume hospitals (HVH) vs Low-volume hospitals (LVH) |
| **O** | Outcome | What are the relevant outcomes? | 30- and 90-day mortality and post-operative outcomes |

**Table S2.** Quality Assessment of the Studies by the Newcastle-Ottawa Scale

| **Included study** | **Selection** | | | | **Comparability** | **Outcome** | | | **Total** |
| --- | --- | --- | --- | --- | --- | --- | --- | --- | --- |
|  | **Representative of the exposed cohort** | **Selection of external control** | **Ascertainment of exposure** | **Outcome not present at the start of the study** |  | **Assessment of outcomes** | **Sufficient follow-up time** | **Adequacy of follow-up** |  |
| Altini M et al., 2015 | * | * | * | * | ** | * | * |  | 8 |
| Arnold BN et al., 2018 | * | * | * | * | ** | * |  |  | 7 |
| Asplund J et al., 2022 | * | * | * | * | ** | * | * | * | 9 |
| Busweiler LAD et al., 2017 | * | * | * | * | ** | * | * |  | 8 |
| Challine A et al., 2021 | * | * | * | * | ** | * |  |  | 7 |
| Choi H et al., 2017 | * | * | * | * | ** | * | * | * | 9 |
| Cibulas MA et al., 2022 | * | * | * | * | ** | * | * |  | 8 |
| Claassen YHM et al., 2018 | * | * | * | * | ** | * | * |  | 8 |
| Claassen YHM et al., 2018 | * | * | * | * |  | * |  |  | 5 |
| Clark JM et al., 2021 | * | * | * | * | * | * |  |  | 6 |
| Coupland VH et al., 2013 | * | * | * | * | ** | * |  |  | 7 |
| D'Journo XB et al., 2021 | * | * | * | * | ** | * | * |  | 8 |
| Diers J et al., 2021 | * | * | * | * | ** | * | * |  | 8 |
| Fumagalli U et al., 2013 | * | * | * | * |  | * | * |  | 6 |
| Gabriel E et al., 2018 | * | * | * | * | ** | * | * | * | 9 |
| Gandjian M et al., 2022 | * | * | * | * | ** | * | * |  | 8 |
| Habbous S et al., 2021 | * | * | * | * |  | * | * | * | 7 |
| Henneman D et al., 2014 | * | * | * | * | ** | * | * | * | 7 |
| Holleran TJ et al., 2022 | * | * | * | * | ** | * | * | * | 9 |
| Hsu PK et al., 2014 | * | * | * | * | ** | * | * |  | 8 |
| Hue JJ et al., 2021 | * | * | * | * | ** | * | * | * | 9 |
| Ichikawa D et al., 2015 |  | * | * | * |  | * | * |  | 5 |
| Ichikawa D et al., 2013 | * | * | * | * |  | * | * |  | 6 |
| Ikoma N et al., 2019 | * | * | * | * | ** | * | * |  | 8 |
| Iwatsuki M et al., 2019 | * | * | * | * | ** | * | * |  | 8 |
| Iwatsuki M et al., 2021 | * | * | * | * | ** | * | * |  | 8 |
| Jafari MD et al., 2013 | * | * | * | * | ** | * |  |  | 7 |
| Ji J et al., 2021 | * | * | * | * | ** | * |  |  | 7 |
| Ju MR et al., 2021 | * | * | * | * | ** | * |  |  | 7 |
| Kennedy GT et al., 2018 | * | * | * | * | ** | * |  |  | 7 |
| Kim BR et al., 2021 | * | * | * | * | ** | * |  |  | 7 |
| Lacueva FJ et al., 2022 | * | * | * | * | ** | * | * |  | 8 |
| Lee HH et al., 2017 | * | * | * | * | ** | * |  |  | 7 |
| Lei LL et al., 2023 |  | * | * | * | ** | * | * |  | 8 |
| Levy J et al., 2020 | * | * | * | * | ** | * |  |  | 7 |
| Markar S et al., 2015 | * | * | * | * | ** | * |  |  | 7 |
| Meng R et al., 2019 | * | * | * | * | ** | * |  |  | 7 |
| Munasinghe A et al., 2015 | * | * | * | * | ** | * | * |  | 8 |
| Murata A et al., 2015 |  | * | * | * | ** | * | * |  | 7 |
| Narendra A et al., 2021 | * | * | * | * | ** | * | * | * | 9 |
| Narendra A et al., 2020 | * | * | * | * | ** | * | * |  | 8 |
| Nimptsch U et al., 2019 | * | * | * | * | ** | * |  |  | 7 |
| Nishigori T et al., 2016 | * | * | * | * | ** | * |  |  | 7 |
| Nuytens F et al., 2022 | * | * | * | * | ** | * |  |  | 7 |
| Oware Adu-Gyamfi K et al., 2020 | * | * | * | * | ** | * |  |  | 7 |
| Patel DC et al., 2020 | * | * | * | * | ** | * | * |  | 8 |
| Salfity H et al., 2019 | * | * | * | * | ** | * | * |  | 8 |
| Shibao K et al., 2020 | * | * | * | * | ** | * |  |  | 7 |
| Smith RC et al., 2014 | * | * | * | * | ** | * | * |  | 8 |
| Ubels S et al., 2023 |  | * | * | * | ** | * |  |  | 6 |
| Voeten DM et al., 2021 | * | * | * | * | ** | * | * |  | 7 |
| Voeten DM et al., 2021 | * | * | * | * | ** | * | * |  | 7 |
| Voeten DM et al., 2021 | * | * | * | * | ** | * | * |  | 7 |
| Voeten DM et al., 2020 | * | * | * | * | ** | * | * |  | 7 |
| Wu JM et al., 2019 | * | * | * | * | ** | * |  |  | 6 |
| Yoshida N et al., 2019 | * | * | * | * | ** | * |  |  | 6 |

**Figure S1.** Funnel plot illustrating publication bias regarding cut-off points for defining high-volume hospitals in the 30-day mortality analysis.

**Figure S2.** Meta-analysis of the relationship between 90-day mortality and hospital volume for esophagectomy and gastrectomy

**
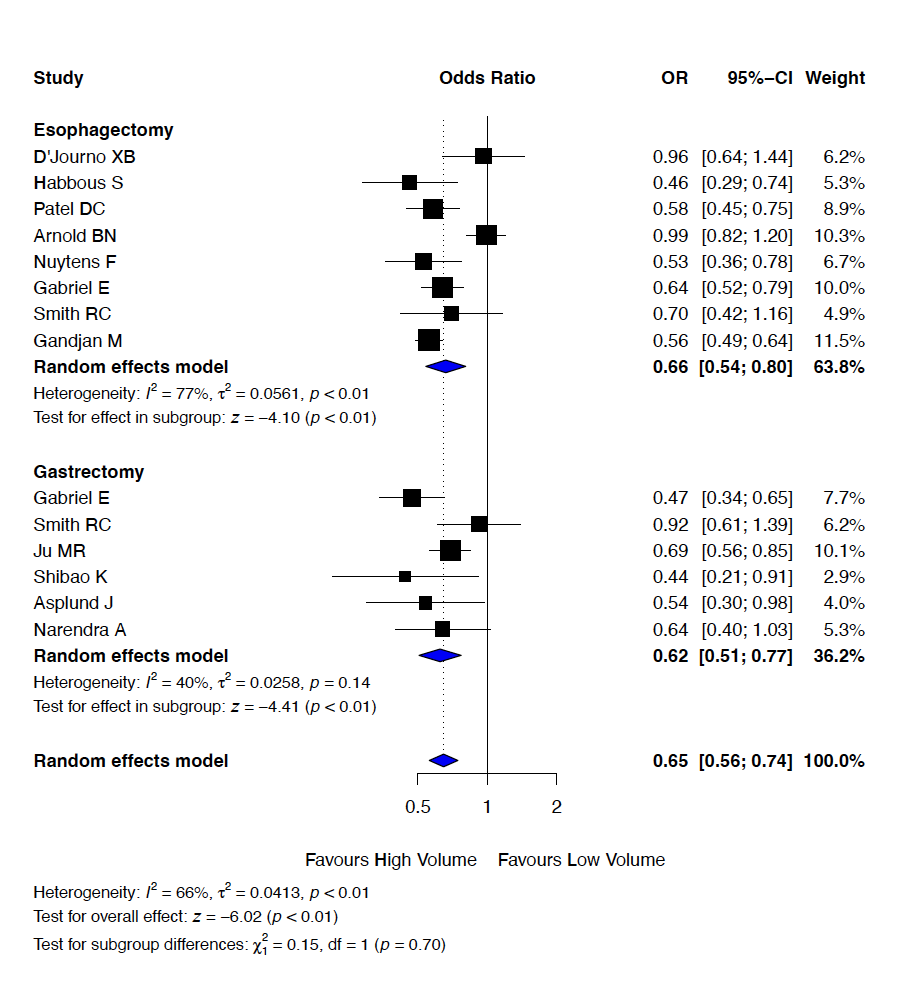
**

**Figure S3.** Meta-analysis of the relationship between postoperative complications and hospital volume for esophagectomy and gastrectomy

**
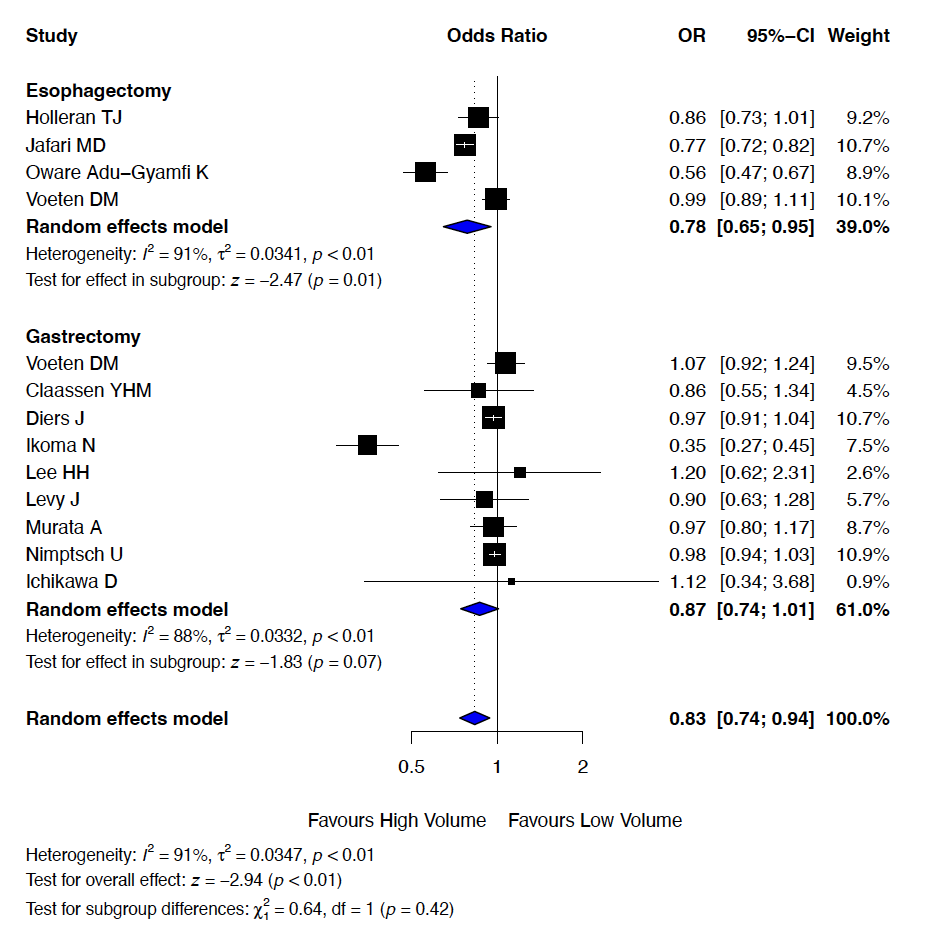
**

**Figure S4.** Meta-analysis of the relationship between pulmonary complications and hospital volume for esophagectomy and gastrectomy**
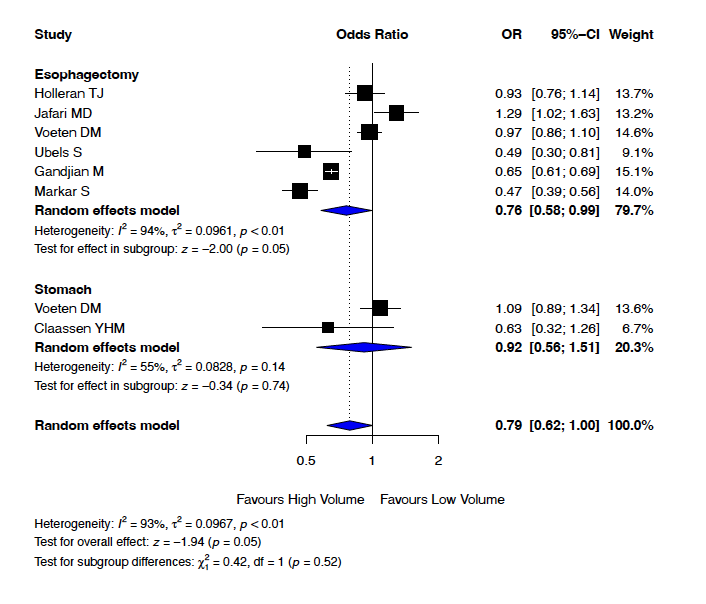
**

**Figure S5.** Meta-analysis of the relationship between anastomotic leakage and hospital volume for esophagectomy and gastrectomy

**
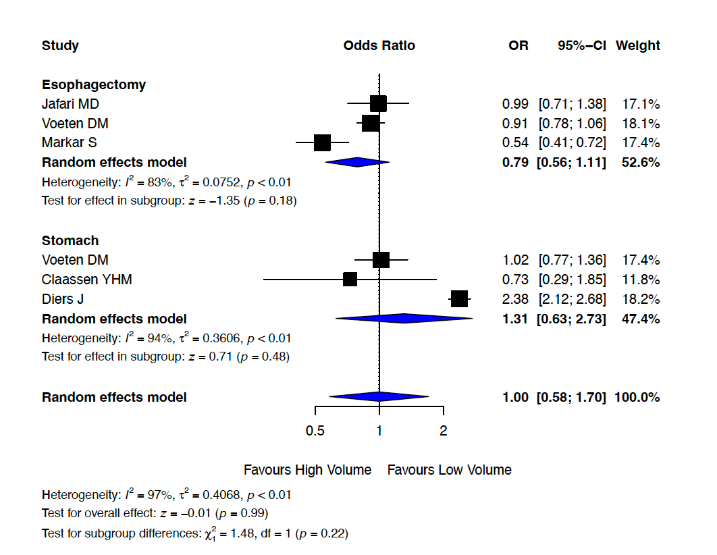
**

**
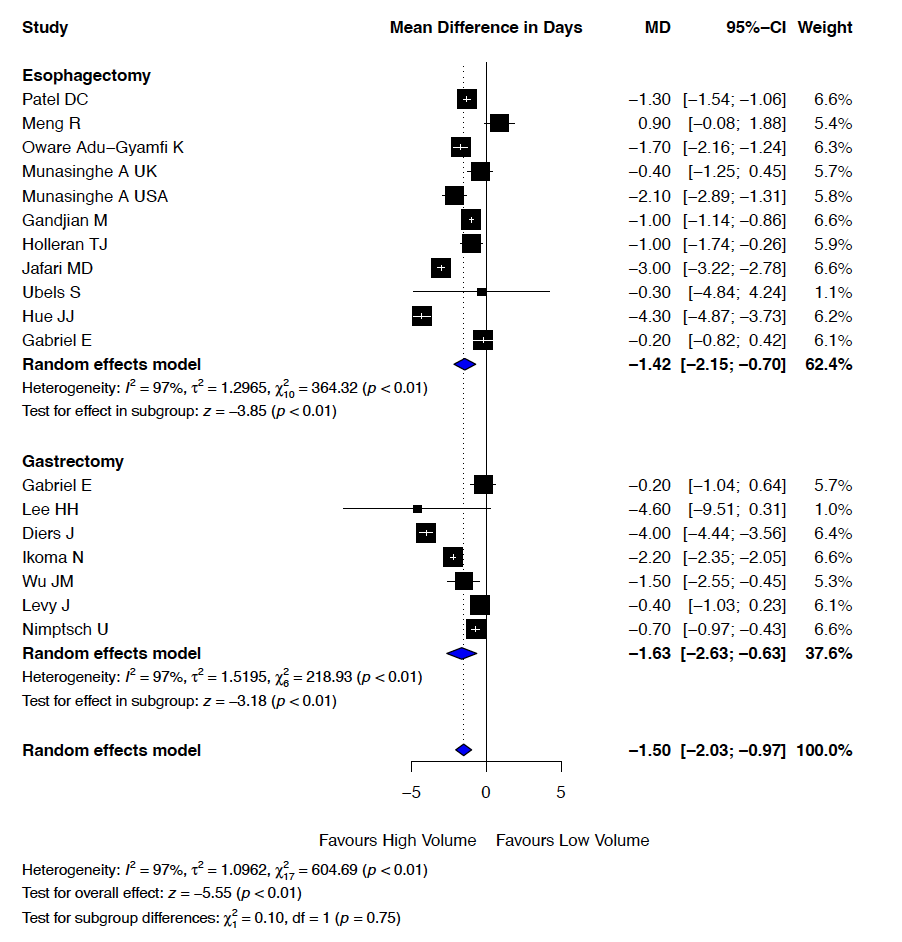
Figure S6.** Meta-analysis of the relationship between length of stay (LOS) and hospital volume for esophagectomy and gastrectomy

**Figure S7.** Meta-analysis of the relationship between readmission rate and hospital volume for **
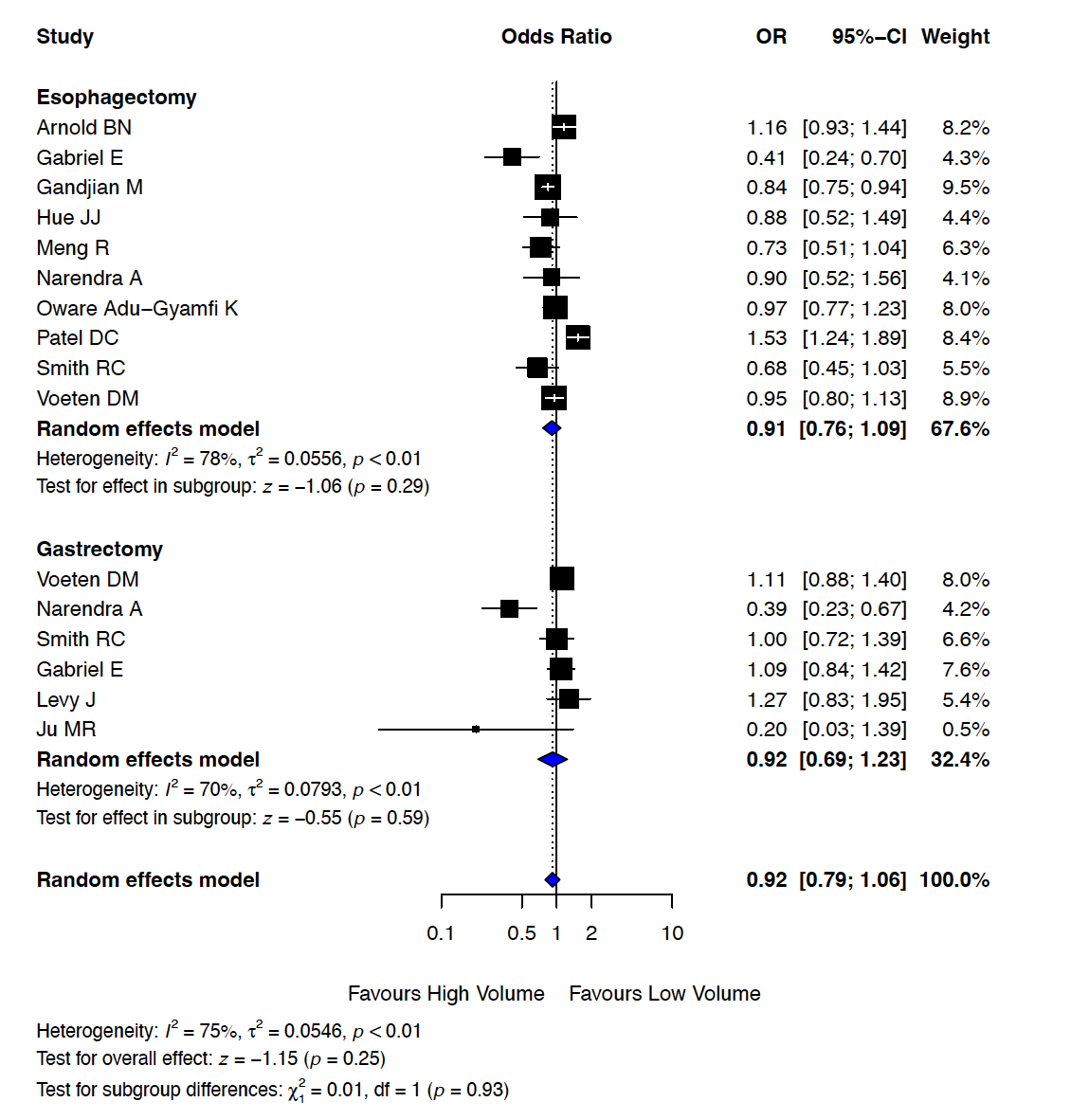
**esophagectomy and gastrectomy


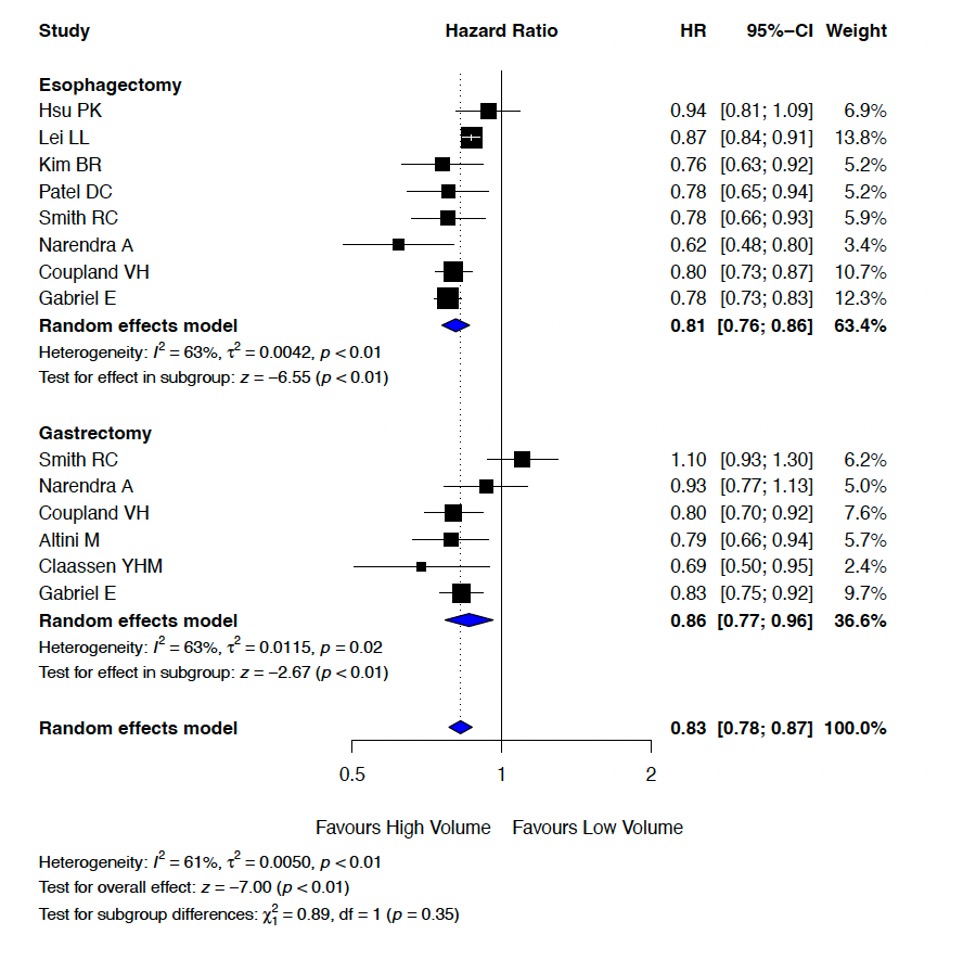
**Figure S8.** Meta-analysis of the relationship between overall survival and hospital volume for esophagectomy and gastrectomy.
